# Transcription factor *ATF3* mediates the radioresistance of breast cancer

**DOI:** 10.1111/jcmm.13688

**Published:** 2018-08-17

**Authors:** Wenyan Zhao, Ming Sun, Shuqiang Li, Zhaofu Chen, Donghua Geng

**Affiliations:** ^1^ Department of General Surgery Shengjing Hospital Affiliated China Medical University Shenyang China; ^2^ Department of Urology Shengjing Hospital Affiliated China Medical University Shenyang China

**Keywords:** *ATF3*, breast cancer, *MCF7* cells, radioresistance

## Abstract

This study was designed to research the influence of activating transcription factor 3 (ATF3) on the radioresistance of breast cancer. ATF3 expression was measured by qRT‐PCR and immunohistochemistry. Cancerous cell lines were cultured in vitro, and the expression of ATF3 was gauged by both qRT‐PCR and Western blot before and after the radiation therapy. Cellular cycle and apoptosis were analysed by flow cytometry. Changes in the expression of corresponding proteins in the downstream pathways were identified by Western blot. Tumour xenograft was used to evaluate the effect of ATF3 in vivo. *ATF3* was observed stronger in breast cancer tissues and cells. After radiation therapy, the expression of *ATF3* in breast cancer cells was up‐regulated. Silencing *ATF3* could promote G2/M phase block, facilitate cell apoptosis and decrease clonogenic survival rate. The overexpression of *ATF3* could curb G2/M‐phase block, cell apoptosis and increase clonogenic survival rate. Overexpression *ATF3* could increase radioresistance by up‐regulating the level of phosphorylation of Akt in the PI3K/Akt signalling pathway. Radioresistance of breast cancer cells could be alleviated by inhibiting the PI3K/Akt signalling pathway. *ATF3* could also promote radioresistance in vivo. *ATF3* gene was able to promote radioresistance of breast cancer cells.

## 1 INTRODUCTION

Breast cancer is generally considered as one of the most common yet fatal cancers among women worldwide.^1^ On the one hand, traditional anti‐cancer therapies, including surgery, chemotherapy and radiation therapy^2^ were proved to have limited effects on breast cancer recovery. When it comes to radiation therapy, especially, the radioresistance of breast cancer cells hinders the cellular apoptosis of breast cancer and decreases the recovery efficiency. On the other hand, although various evidence have all come to indicate that activating transcription factor 3 (*ATF3*) may be high expressed in various cancer cells,^3^ there have been no studies investigating the clinical significance of ATF3 in human breast cancer until recently.^4^

*ATF3* is a transcription factor from the ATF/CREB family.^5^ Overpowering evidence proved that ATF3 translated by an immediate early gene and its expression is weak in various cells. But *ATF3* expression can be triggered by multiple cellular signals.^6^ According to existing investigations, *ATF3* is supposed to be a crossroad of the cellular response network and *ATF3* have been proved to take a place on canceration course of breast epithelial cells.^7^ Furthermore, it promotes motility of breast cells and metastasis from epithelial to mesenchymal by TGF‐β signalling.^8^

The PI3K/Akt pathway is involved in many cellular functions, including protein synthesis, cell cycle progression, cell survival, cell apoptosis, angiogenesis and drug resistance.^9^ Multiple biological processes, such as cell proliferation, cell metabolism and cell survival, are all regulated by Akt.^10^ The PI3K/Akt signal pathway mediates cell survival by promoting aerobic glycolysis.^11^ Most of the cancer cells produce abundant lactate to supply energy, but it is inefficient to generate ATP. This phenomenon is regarded as aerobic glycolysis.^12^ Akt can mediate various steps of glycolysis by post‐transcriptional mechanisms which contain promoting hexokinase activity and up‐regulating expression of glucose transporter Glut1.^13^ Recent report showed that increased expressions of glucose transporter Glut1 and lactate were examined in acquired radioresistant cells.^14^ Shimura et al. unearthed that inhibition of glycolysis could control required tumour cell radioresistance. In this study, we would like to investigate the effect of *ATF3* in breast cell radioresistance by controlling the production of pAkt and ATF3.

Radiosensitivity of breast cancer cells may be altered by the reversible PI3K inhibitor LY294002, which inhibits certain mammalian PI3Ks by covalent or non‐covalent modification of a critical lysine residue in their phosphotransferase domains.^15^ On account of the presence of the COOH‐terminal sequence homology among the PI3K, we can draw a conclusion that the PI3K/Akt signalling pathway may also be sensitive to the inhibition of LY294002.^16^ In a recent study of non‐small cell lung cancer, it was found out that high levels of PI3K/Akt activity increased the radioresistance of these cells and suppressed the radiation‐induced cell apoptosis; but once the cells were treated with LY294002, sensitivity to radiation therapy was restored.^17^ The results of these studies all suggested that modulation of PI3K/Akt activity in cancer cells may alter the sensitivity of the cells to conventional radiation therapy.

In accordance with all the reports above, we have decided to disclose the relationship between the radioresistance of breast cancer cells and the expression of *ATF3* in the PI3K/Akt signalling pathway.

## 2 MATERIALS AND METHODS

### 2.1 Patients and tissue specimens

Sixty specimens of breast cancer patients who had gone through radiotherapy and been confirmed pathologically were collected from Shengjing Hospital Affiliated China Medical University (from June 2015 to May 2016). All the breast cancer tissues and paracancerous tissues of patients were placed immediately in liquid nitrogen and kept for long‐time preservation in −80°C to be measured. All participates involved in this study have signed the consent informs. Clinicopathological features of breast cancer patients were listed in Table S1.

### 2.2 Microarray analysis

Differentially expressed genes were hybridized and selected by Human LncRNA Microarray V3.0 (GSE59732, https://www.ncbi.nlm.nih.gov/geo/query/acc.cgi?acc=GSE59732). mRNA expressions of 96 breast cancer cell samples were downloaded from Gene Expression Omnibus (GEO, https://www.ncbi.nlm.nih.gov/geo/) public database. The data of T47D cells before or after irradiation were analysed by R project (https://www.r-project.org/) and log_2_ (Fold Change) >2 and *P* < .05 were identified as our filtration criteria.

### 2.3 Cell culture

Breast cancer cell lines T47D, ZR751, MCF7, HBL10, SUM159 and normal human mammary epithelial cell line MCF10A were all obtained from the Department of Cell Biology, School of Basic Medicine Peking Union Medical College, Institute of Basic Medical Sciences Chinese Academy of Medical Sciences. After being diluted to 10 mL by 10% foetal bovine serum medium, cell lines were placed in a 37°C constant temperature CO_2_ incubator for normal subculture and cell growth was observed.

### 2.4 Cell transfection

BLOCK‐iTTM RNAi Designer (http://rnaidesigner.thermofisher.com/rnaiexpress/) was employed to design interference nucleotide sequence (si*ATF3,* sh*ATF3*), and independent nucleic sequence with the same base number was designed and synthesized as negative control (siNC, shNC) (relative sequence, Table S2). Si*ATF3* and siNC were used for in vitro experiments, while sh*ATF3* and shNC were used for in vivo experiments. When the sequence was transformed into competent cells for amplification, positive clones were selected and recombinant plasmid was extracted and underwent sequencing identification. Liposome transfection, in accordance with Lipofectamine™ 2000 (Invitrogen, CA, USA) was employed to transfect. Transfection efficiency was observed at the 24th hour and the 48th hour after cell culture. Plasmid LZRS‐IRES‐zeo‐ATF3 was built by connecting carrier LZRS‐IRES‐zeo and *ATF3* cDNA (*ATF3*) and blank control group LZRS‐IRES‐zeo (NC) was set up.

### 2.5 Immunohistochemistry

The paraffin‐embedded pathological sections were waxed, dehydrated by gradient alcohol. The endogenous peroxidase was eliminated and the antigen was repaired. 100 μL antigen of ATF3 (rabbit anti‐human, 1:800, Abcam, USA) was injected to be incubated overnight. HRP‐labelled goat anti‐rabbit IgG (ZSGB‐BIO, Beijing, China) was incubated, taken out 40 minutes later and was washed with phosphate buffered saline (PBS) thrice. DAB chromogenic agent was used for colour development for about 20 seconds. After haematoxylin restraining for 1 minute, it was dehydrated with gradient alcohol, dried and mounted. The expression level of ATF3 was evaluated by semi‐quantitative analysis.

### 2.6 qRT‐PCR

RNA primers were designed and synthesized (Table S3 for primer sequences, synthesized by Sangon Biotech, Shanghai, Co., Ltd. China). Total cellular RNA was extracted using Trizol method. Then the purity and concentration of total RNA were tested. Reverse transcription was proceeded using Revert Aid First Strand cDNA Synthesis Kit (Thermo Fisher Scientific, MA, USA) according to the Maxima SYBR Green qPCR Master Mix (2X) kit instructions (Thermo Fisher Scientific). The corresponding reaction system was added under dark condition, PCR amplification was implemented with β‐actin used as an internal reference, the experimental setup procedures were as follow: 95°C, 5 minutes; 95°C, 5 minutes; 40 cycles of reaction; 95°C,15 seconds; 60°C, 30 seconds, the results of the experiment were recorded after the cycles were completed. The results were analysed with ABI Step One Software V 2.1 software (Applied Biosystems, MA, USA), and the relative expression of genes was analysed by the formula $2^{-\Delta \Delta C_{t}}$.

### 2.7 Western blot

In order to extract the total protein, RIPA lysis was employed to lyse the cells. The total protein concentration of each group was detected by bicinchoninic acid (BCA) method. SDS‐PAGE gel was prepared for sample electrophoresis. The polyvinylidene fluoride film (PVDF) was removed and placed in a sealing fluid containing 3% of albumin from bovine serum (BSA). The primary antibody (ATF3 rabbit anti‐human, 1:5000, ab207434 Abcam; β‐catenin, 1:5000, ab8227, Abcam; pAkt, 1:5000, ab38449 Abcam; Akt, 1:5000, ab81283, Abcam; caspase3, 1.5 μg/mL, ab13585, Abcam) was diluted by the Tris‐buffered saline solution with tween (TBST), then blocked overnight in a shaker at 4°C. PVDF film was taken out and washed three times with TBST. Goat anti‐rabbit HRP secondary antibodies (ab6741, Abcam, USA) were diluted 1:5000. Both were blocked inconstant temperature shaker for 4 hours and washed three times with TBST. After darkroom exposure, development, cleaning and fixing, stripe accumulative optical density was analysed by ImageJ software (Version 1.48u, Bethesda, USA) semi‐quantitative analysis and relative expressions of target proteins were calculated, respectively.

### 2.8 Radiation therapy

Different doses (2 Gy/min, 4 Gy/min, 6 Gy/min or 8 Gy/min) of radiation therapy were exerted on the cells with Philips RT250 (Kimtron Medical, CA, USA).

### 2.9 Clonogenic survival analysis

The cells in the logarithmic growth phase were extracted, digested into individual cells with trypsin solution, and then added RPMI1640 medium. 1000 cells were spread evenly with the appropriate concentration of the cell suspension in a petri dish at 37°C, 5% CO_2_. When the cells were visible to the human eye, the culture was terminated. The cells were radiated with Caesium‐137 Mark‐I irradiator (JL Shepherd, CA, USA) of 2 Gy, 4 Gy, 6 Gy or 8 Gy for 24 hours. Media was removed and replaced 24 hours after irradiation. The cells were allowed to grow into colonies over a period of 10‐14 days or until the control plates grew visible colonies. Cells were then washed with PBS, followed by fixed with 10% methanol and 10% acetic acid for 10 minutes. The cells were dyed for 10 minutes with 0.4% crystal violet solution and dried in the air after the removal of the stain. Clones were calculated using ColCounte colony counter (Oxford Optronix Ltd, Abingdon, UK), the sensitivity was confirmed when the cell number exceeded 50. The following formula was used to calculate the Plating Efficiency (PE): PE = clone number/inoculation number. The comparison was standardized to the negative control group. The survival fraction (SF) was calculated using the following formula: SF = clone number/inoculation number × PE.

### 2.10 Cell apoptosis analysis

Cell apoptosis was analysed by Cell Cycle and Apoptosis Analysis Kit (Beyotime, Jiangsu, China). Breast cancer cells were collected at the 36th hour after the transfection and then treated with radiotherapy in three different groups: the group adding only PI, the group adding only Annexin V‐FITC and the blank control group with neither of the two dyes. After centrifugation, cells were collected into EP tubes and the number of cells per tube was no less than 1 × 10^6^. The cells were washed twice with PBS and centrifuged at constant temperature at 1000 rpm for 5 minutes. After the supernatant was discarded, the cells were added to 500 μL binding buffer and resuspended. In turn, 5 μL Annexin V‐FITC and 5 μL PI were added to the cells, gently mixed, placed on ice in the dark for 15 minutes, and then examined by flow cytometry method (FCM).

### 2.11 Caspase 3 activity detection

Pentose nucleic acid (PNA) was diluted with the standard diluent of Caspase 3 Activity Assay Kit (Beyotime, Jiangsu, China). The standard curve was drawn by a microplate reader (Thermo Fisher Scientific, MA, USA) with PNA concentration as *x*‐axis and light absorption value as *y*‐axis. Breast cancer cells were collected at the 36th hour after the transfection, and cell concentration was adjusted to 2 × 10^6^ cells per group. The cells were centrifuged at 4°C for 5 minutes (1000 rpm) with supernatant discarded, and step repeated. The cell lysis solution was added. Afterwards, cells were placed in ice for 15 minutes after resuspension and centrifuged at 4°C for 15 minutes (14 000 *g*). 10 μL of supernatant was extracted and transferred to the pre‐cooled EP tube, 80 μL of detection buffer and 10 μL of Ac‐DEVD‐PNA were added and mixed evenly. The solution was incubated overnight at 37°C and the value of OD_490_ was detected by the microplate reader. The activity of caspase 3 protein was semi‐quantitatively measured by Western blot.

### 2.12 Cell cycle analysis

The test is implemented following the instructions of Cell Cycle and Apoptosis Analysis Kit (Beyotime, Jiangsu, China). The radiation therapy was carried out 36 hours after the cell transfection. The cells were digested by trypsin, collected by centrifuge (1000 rpm) for 5 minutes and the culture medium was discarded. Then the cells were rinsed with cold PBS and centrifuged (1000 rpm) for 5 minutes. 1 mL of cells was added in the cooled 70% alcohol and placed at 4°C overnight. After that, the cells were washed with PBS twice and added with 100 μL of RnaseA and incubated at 37°C water for 30 minutes. 50 μL of PI was then added to the cell mixture and the solution was kept from light for 1 hour. Finally, the cell cycle was detected by flow cytometry method (FCM).

### 2.13 Tumour xenograft

All animal experiments were performed according to the National Institutes of Health guide for the care and use of Laboratory animals. All procedures were approved by the Institutional Animal Ethical Committee of Shengjing Hospital Affiliated China Medical University. 40 female BALB/c nude mice (5‐6 week) were obtained from the laboratory animal centre of Shengjing Hospital Affiliated China Medical University. All mice were hypodermic injected 10^7^ MCF7 or SUM159 cells on their back. After tumours reached about 100 mm^3^, mice were randomly divided into 8 groups (5 mice/group). For radiation groups, 2 Gy of local radiation were delivered to mice every day. Tumour volume was measured by the caliper per 5 days and calculated using the formula: V = (length × width^2^)/2. After 30 days, mice were killed by inhalation CO_2_ and tumor tissues were excised and weighed.

### 2.14 Statistical analysis

GraphPad Prism 6.0 software (Version 6, CA, USA) was used for statistical analysis. The differences between the two samples were measured by the *T*‐test, and the differences among multiple samples were analysed using the one‐way analysis of variance. When *P* was <.05, the difference was considered statistically significant.

## 3 RESULTS

### 3.1 ATF3 was highly expressed in breast cancer after radiotherapy

**Figure 1:**
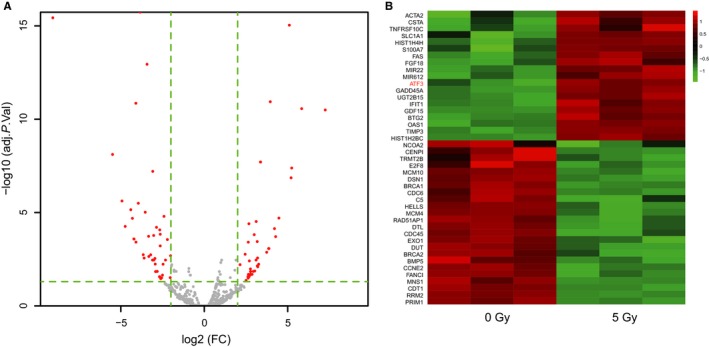
*ATF3* was in a high expression in multiple breast cancer cell lines. A, Volcano plots, differentially expressed genes were analysed by microarray; B, Heat map showed that *ATF* was up‐regulation when treated with 5 Gy radiotherapy

According to the microarray analysis, 44 genes were revealed significant up‐regulation in radiated breast cancer cell samples under the condition that the false positive rate was less than 0.01 and fold change >2 (Figure 1A,B). Finally, *ATF3* which was strongly expressed in multiple breast cancer cell samples were chosen for next experiments.

### 3.2 ATF3 was up‐regulated both in breast cancer tissues and cells

**Figure 2:**
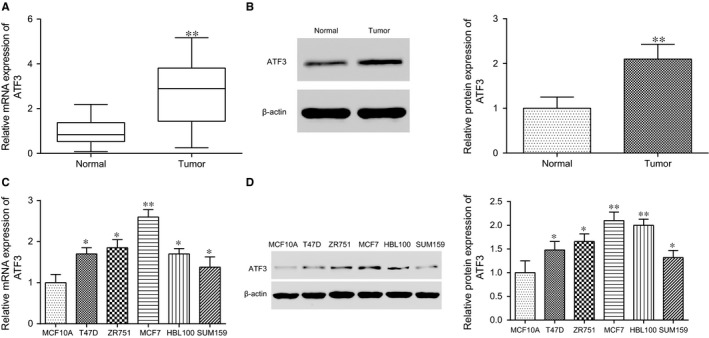
*ATF3* was highly expressed in both breast cancer tissues and cell lines. A, *ATF3* mRNA expression was up‐regulation in breast cancer tissue; B, The relative protein expression of ATF3 in breast cancer tissues was up‐regulated; C, The mRNA expression level of *ATF3* increased in different cell lines; D, The relative protein expression of ATF3 in multiple cell lines rose. *P < .05, **P < .01, compared with normal or MCF10A

To observe the expression of *ATF3* in breast cancer tissues, 60 clinical samples collected from Shengjing Hospital Affiliated China Medical University were examined by qRT‐PCR. It was found out that the expression level of *ATF3* in breast cancer tissues which had received radiotherapy was strongly higher than that in adjacent tissues (*P* < .01, Figure 2A,B). After the analysis of *ATF3* expression level in different cell lines, it was found out that the mRNA expression level of *ATF3* was also remarkably higher in breast cancer cell lines T47D, ZR751, MCF7, HBL100 and SUM159 than that in normal human mammary epithelial cell line MCF10A (all *P* < 0.05, Figure 2C). The protein expression of ATF3 was assessed by Western blot and the protein expression of ATF3 was also markedly up‐regulated in breast cancer cell lines (all *P* < .05, Figure 2D).

### 3.3 ATF3 expression increased in breast cancer cells after the radiation therapy

**Figure 3:**
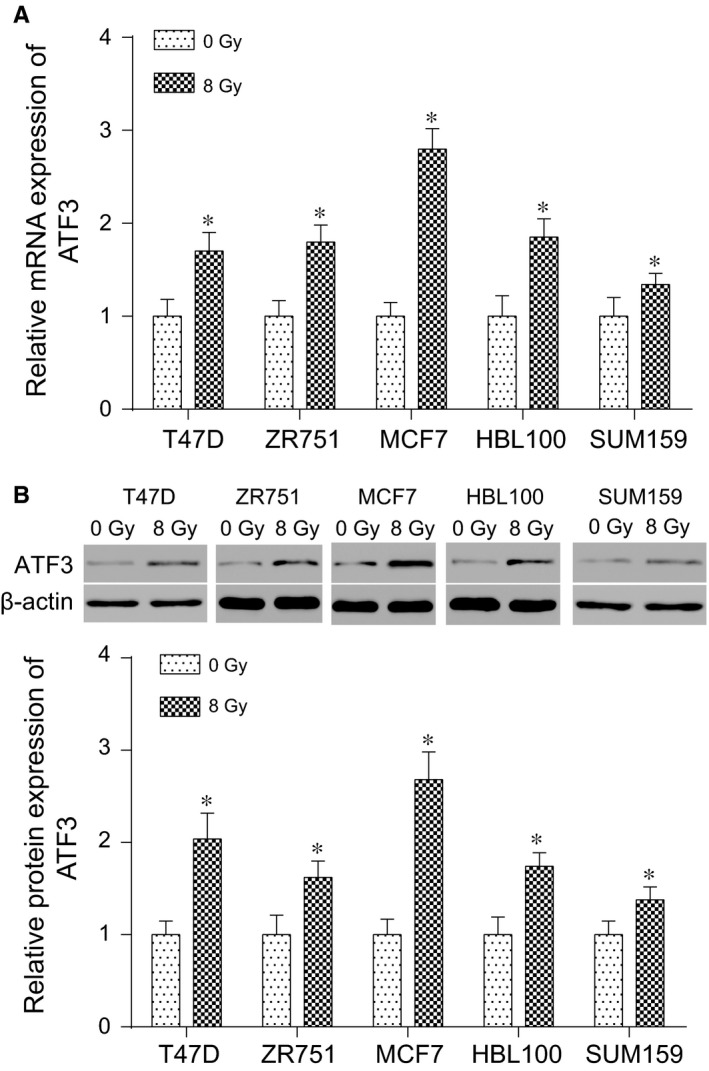
*ATF3* production was increased after ionizing radiation. A, Relative mRNA expression of *ATF3* rose in different cell lines after the radiation therapy. B, Relative protein expressions of *ATF3* in different cell lines before and after the radiation therapy were measured by Western blot. *P < .05, compared with 0 Gy group

To simulate the clinical situation of radiation resistance, T47D, ZR751, MCF7, HBL100 and MCF7 cell lines were treated with radio ionizing radiation. The result showed that both mRNA and protein expressions of ATF3 in all cell lines were increased (*P* < 0.05, Figure 3A,B). As the most outstanding differences observed in the MCF7 and SUM159 cell lines, radioresistant MCF7 was used to carry out the knockout experiments whereas radiosensitive cell line SUM159 was used to carry out the overexpression experiments in the subsequent experiments.

### 3.4 The expression quantity ATF3 in MCF7 and SUM159 cells

After the MCF7 was transfected with siATF3, qRT‐PCR and Western blot were utilized to measure the expression of ATF3. Depending on the detection result, compared with the negative control group siNC, the expression level of ATF3 in the siATF3 group was significantly reduced (all *P* < .05, Figure S1A‐S1B), which proved that the interference was effective. After the SUM159 cell line was transfected with the LZRS‐IRES‐zeo‐*ATF3*, the ATF3 mRNA and protein expressions of the LZRS‐IRES‐zeo‐*ATF3* both significantly rose (*P* < .05, Figure S1C‐S1D) in contrast to the negative control group LZRS‐IRES‐zeo, which proved that the overexpression was effective.

### 3.5 ATF3 could promote the radioresistance of breast cancer cell

After the MCF7 cell line was transfected with siATF3, the survival rate of the cell clone in siATF3 group was significantly cut down (all *P* < .05, Figure S2A) compared with the negative control group. After the SUM159 cell was transfected with the expression vector LZRS‐IRES‐zeo‐*ATF3*, the survival rate of the LZRS‐IRES‐zeo‐*ATF3* cell clone was notably improved compared with the negative control (all *P* < .05, Figure S2B). It could be possible to conclude that the low expression of *ATF3* could alleviate the radioresistance of breast cancer cells.

### 3.6 Overexpression ATF3 could lessen apoptosis rate induced by radiation

**Figure 4:**
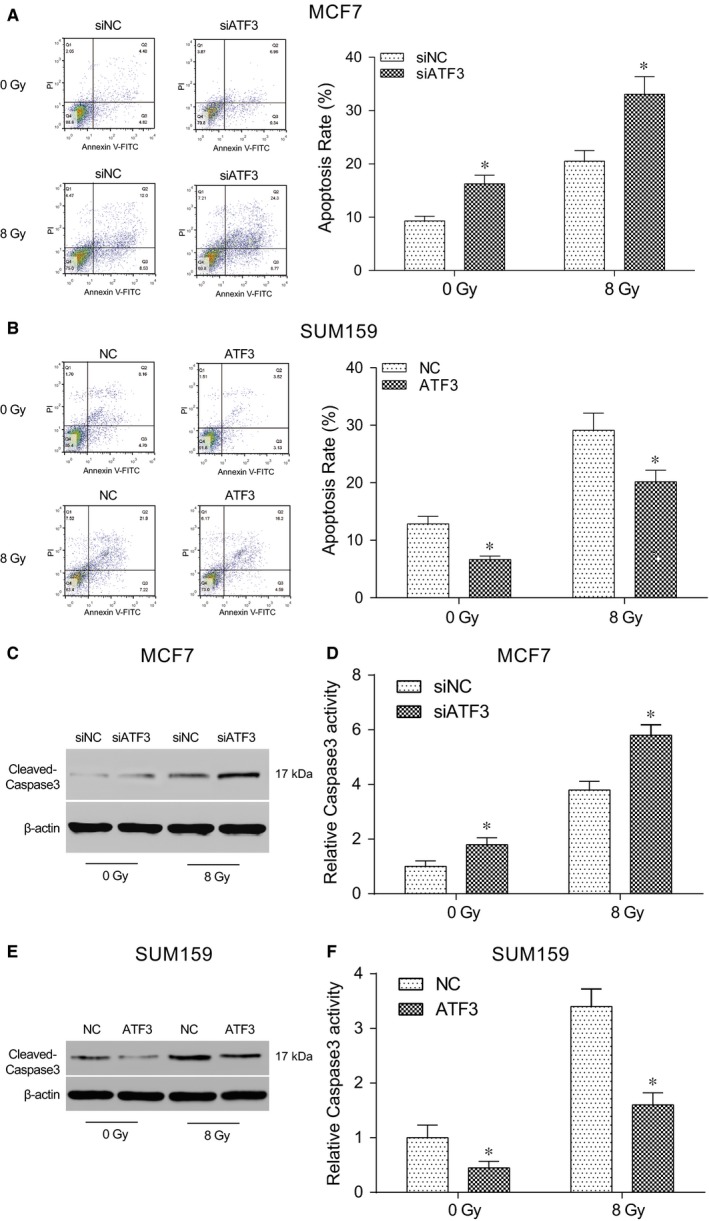
Apoptosis rate of breast cells significantly rose after treating with irradiation. A, The apoptosis rate was remarkably improved after the radiation therapy and was evidently higher than that of the negative control group; B, The apoptosis rate was remarkably increased after the radiation therapy and was evidently lower than that of the negative control group; C, Apoptosis‐related protein cleaved‐caspase3 was up‐regulation after the radiation therapy in MCF7 cells transfected with siATF3; D, The activity of caspase3 was significantly improved after the radiation therapy and was evidently higher than that of the negative control group in MCF7 cells; E, Apoptosis‐related protein cleaved‐caspase3 was also up‐regulation after the radiation therapy in SUM159 cells transfected with LZRS‐IRES‐zeo‐*ATF3*; F, The activity of caspase3 was significantly improved after the radiation therapy and was evidently lower than that of the negative control group. siNC: cells transfected with independent nucleic sequence with the same base number. si*ATF3*: cells transfected with interference nucleotide sequence. NC: cells transfected with empty plasmid LZRS‐IRES‐zeo. *ATF3*: cells transfected with plasmid LZRS‐IRES‐zeo‐ATF3. *P < .05, compared with NC or siNC group

The results of flow cytometry showed that, compared with the negative control group, the frequency of apoptosis rate significantly rose after the MCF7 cell line was transfected with siATF3 (*P* < .05, Figure 4A). And compared with the negative control group, the apoptosis rate was significantly reduced after SUM159 cell line was transfected with LZRS‐IRES‐zeo‐*ATF3* (all *P* < .05, Figure 4B). As shown in Figure 4C‐F, cleaved‐caspase3 protein expression level detected by Western blot was markedly increased after silencing the expression of ATF3. Moreover, the caspase3 activity was further improved after the radiation therapy whereas the activity of caspase3 reduced after the radiation therapy and infected with the overexpression of *ATF3*. All the differences were statistically significant (*P* < .05). It could suggest that the low expression of *ATF3* could improve the percentage of radiation‐induced cell apoptosis.

### 3.7 Silence of ATF3 reduced the cell number in G2/M phase

**Figure 5:**
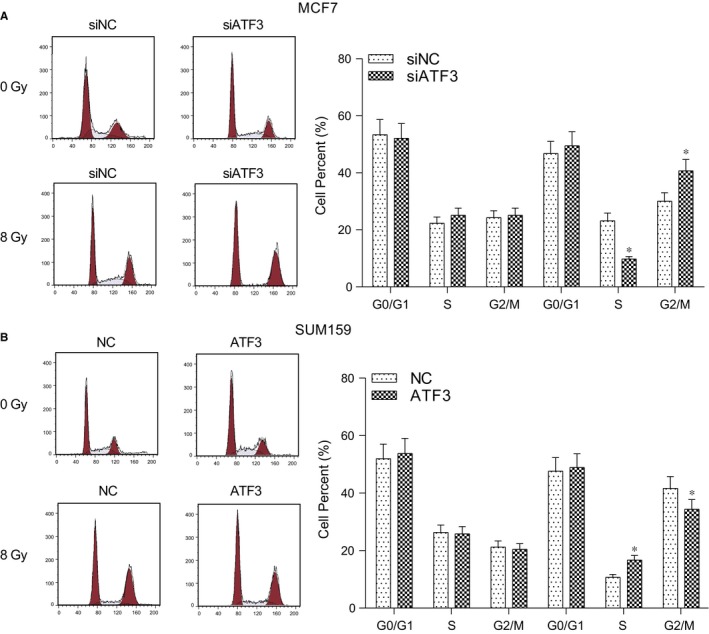
Expression of *ATF3* affected the changes in the radiation‐induced cell cycle. A, After the radiation therapy, the G2/M phase cell number increased markedly and G2/M phase cell number in the siATF3 group was remarkably larger than that in the negative control group. Cells in S phase had opposite trend; B, After the radiation therapy, the G2/M phase cell number increased markedly and G2/M phase cell number in the LZRS‐IRES‐zeo‐*ATF3* group strongly decreased than that in the negative control group. Cells in S phase had opposite trend. siNC: cells transfected with independent nucleic sequence with the same base number. si*ATF3*: cells transfected with interference nucleotide sequence. NC: cells transfected with empty plasmid LZRS‐IRES‐zeo. *ATF3*: cells transfected with plasmid LZRS‐IRES‐zeo‐ATF3. *P < .05, compared with NC or siNC group

The results of flow cytometry exhibited that after the radiation therapy, the cell rate of G2/M phase obviously augmented whereas cells in S phase were obviously shrunken in the siATF3 group in comparison with the negative control group (*P* < .05, Figure 5A). And compared with the negative control group, the G2/M phase cell rate of the LZRS‐IRES‐zeo‐*ATF3* group was significantly lessened whereas cells in the S phase notably increased (*P* < .05, Figure 5B). Therefore, it could indicate that the low expression of *ATF3* contributed to G2/M phase block in the cell cycle.

### 3.8 ATF3 facilitated the radioresistance of breast cancer cells through the PI3K/Akt signalling pathway

**Figure 6:**
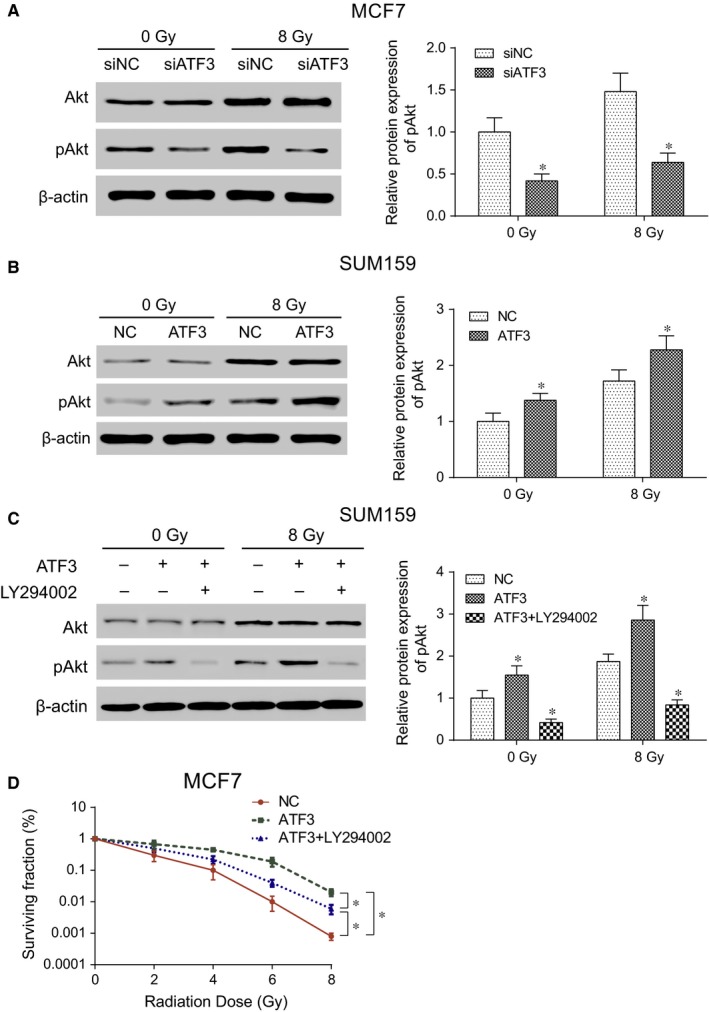
Radiation therapy induced *ATF3* expression leading to the activation of PI3K/Akt signalling pathway. A, The Akt phosphorylation process was inhibited after silencing *ATF3* in the MCF7 cell line; B, The Akt phosphorylation process was improved after the *ATF3* overexpression in the SUM159 cell line; C, The Akt phosphorylation process was inhibited after the *ATF3* overexpression in SUM159 cell line treated with inhibitor LY294002; D, The overexpression of *ATF3* could accelerate the survival rate of breast cancer cells whereas inhibitor LY204002 could inhibit the survival rate of breast cancer cells. siNC: cells transfected with independent nucleic sequence with the same base number. si*ATF3*: cells transfected with interference nucleotide sequence. NC: cells transfected with empty plasmid LZRS‐IRES‐zeo. *ATF3*: cells transfected with plasmid LZRS‐IRES‐zeo‐ATF3. *ATF3*+ LY294002: cells treated with both transfection of plasmid LZRS‐IRES‐zeo‐ATF3 and PI3K inhibitor LY294002. *P < .05, compared with NC or siNC group

Results of Western blot demonstrated that after the radiation therapy, the protein expression level of radioresistance‐related key protein pAkt in siNC group and siATF3 group of MCF7 cell line both increased significantly. But pAkt protein expression was suppressed after silencing the *ATF3* expression (*P* < .05, Figure 6A). On the contrary, the overexpression of *ATF3* led to an increased level of pAkt protein and the increase was even higher after the radiation therapy (*P* < .05, Figure 6B). The results suggested that *ATF3* could augment the radioresistance of breast cancer cells by affecting the related proteins expressions in the PI3K/Akt signalling pathway. As Akt is one of the PI3K pathway downstream regulating elements, LY294002 was utilized to interfere with activation of the pathway in the SUM159 cell line. The effects of PI3K pathway inhibitor LY294002 on radiation‐induced Akt phosphorylation level in cell lines with *ATF3* overexpression were then explored using the clonogenic survival assay and Western blot. The results exhibited that the radiation‐induced phosphorylation was significantly inhibited by LY294002 in the SUM159 cell line (all *P* < .05, Figure 6C). It demonstrates that the effects of *ATF3* expression and of radiation therapy on Akt phosphorylation depend on the activity of upstream PI3K pathway. The clonogenic survival assay showed that the survival rate of cells was significantly reduced after adding the inhibitor LY294002 (all *P* < .05, Figure 6D) compared with the *ATF3* overexpression group, suggesting that the breast cancer radioresistance established by the *ATF3* overexpression can be alleviated by inhibiting the PI3K/Akt signalling pathway.

### 3.9 ATF3 enhanced the radioresistance of breast cancer in vivo

**Figure 7:**
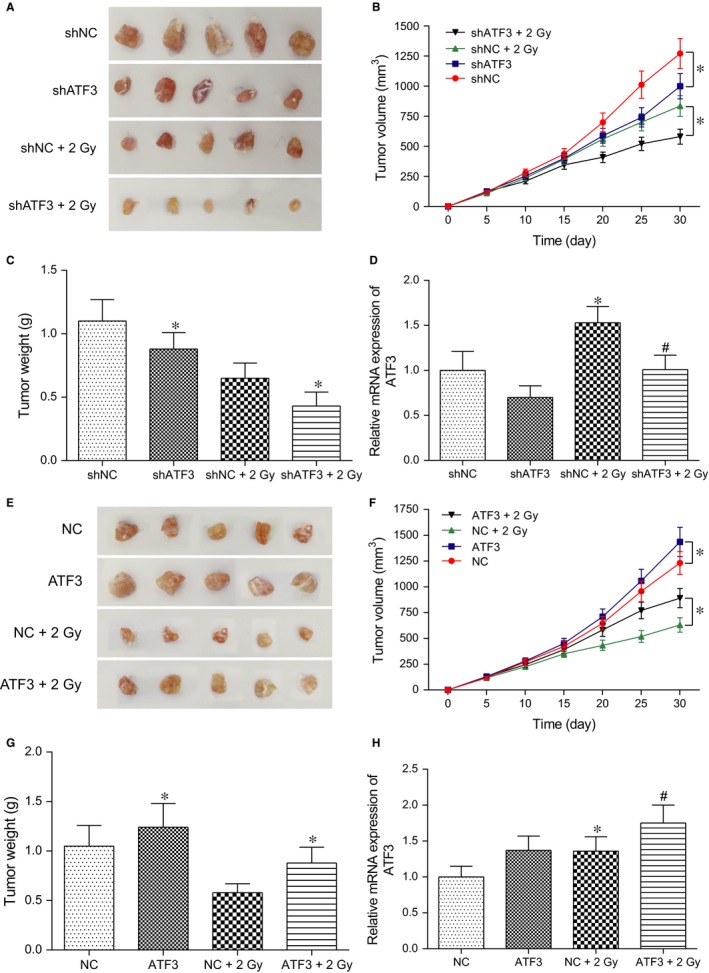
ATF3 facilitated the radioresistance of breast cancer in vivo. A&E, The photo of the tumours; B&F, The volume curve of the tumours; C&G, The weight of the tumours after 30 days; D&H, The relative expression of *ATF3* in the tumour. *P < .05, compared with NC or NC+2 Gy group. ^#^ *P* < .05, compared with siATF3 or ATF3 group

To further explore the effect of ATF3 in vivo, tumour xenograft experiments were performed. After 30 days, mice were killed and tumour tissues were excised (Figure 7A,E). The results showed that overexpression of *ATF3* could promote the growth of tumours (*P* < .05, Figure 7B,C), while silence of *ATF3* could inhibit the development of tumours (*P* < .05, Figure 7F,G). In addition, *ATF3* attenuated the effect of irradiation in vivo (*P* < .05, Figure 7B,C), while silence of *ATF3* enhanced the sensitivity of irradiation of breast cancer (*P* < .05, Figure 7F,G). On the other hand, qRT‐PCR was carried out to detect the level of *ATF3* in tumour. It revealed that the level of *ATF3* was up‐regulated followed irradiation compared with control group (*P* < .05, Figure 7D,H). All these indicated that ATF3 was able to enhance radiotherapy tolerance in vivo as well.

## 4 DISCUSSION

In this present study, we detected that breast cancer radioresistance was enhanced when the *ATF3* expression was up‐regulated, resulting in less breast cancer cell apoptosis, decreased G2/M phase block and more activated PI3K/Akt signalling pathway. On the contrary, if *ATF3* expression in breast cancer cells was silenced by the siATF3 transfection or was suppressed by the signalling pathway inhibitor LY204002, breast cancer radioresistance could be relieved to a great extent and the radiation therapy efficiency can be also improved. Overwhelming evidence showed that *ATF3* was highly expressed and was closely related to cell migration in breast cancer.^6^, ^18^ Gokulnath et al. found that silencing of *ATF3* expression could also decrease the production of MMP‐13 and Runx2 genes related to invasive and metastatic.^18^ However, previous studies revealed that the ethyl acetate fraction (EAFAD‐B) from Abeliophyllum distichum Nakai could resist cancer by up‐regulating *ATF3* repression in colorectal cancer.^19^ In our study, differentially expressed genes were analysed by microarray. As it was found out, *ATF3* expression was significantly higher in breast cancer tissues and cells after radiation therapy. Therefore, the following experiment was focused on the relationship between *ATF3* expression and radioresistance of breast cancer. Previous studies concentrating on the relationship between differentially expressed genes and breast cancer cell radioresistance, such as one studying the expression of ABT‐737, Bcl‐2 and Bcl‐xL in breast cancer,^20^ have all enlightened us to investigate the function of some protein in cell acquired radioresistance. The present results showed that *ATF3* expression increased markedly compared with controls after it was ionized radiation in various cell lines. These findings indicated that *ATF3* had some inevitable relationships with radioresistant of breast cancer cell. So a series of experiments were performed to validate the association of *ATF3* with cell radioresistance in MCF7 and SUM159 cell lines. Recent study which was focused on the relationship between Δ2‐troglitazone (Δ2‐TGZ) and apoptosis in breast cancer cell discovered that cell apoptosis rate increased when *ATF3* was highly going up.^21^ The similar result was revealed in our research that apoptosis rates both increased in MCF7 and SUM159 cells treated with radiation but overexpression *ATF3* could slow it down. Furthermore, it was discovered that siATF3 lowers radioresistance in breast cancer cell by contributing to the G2/M phase block and the G2/M phase block remains longer when radiation dosage increased. Investigations on the connection between Runx2 and ATF3 indicated analogical findings.^18^ However, potential molecular mechanisms of ATF3‐medicated cell radioresistance were still not clear.

Multiple cellular processes including cell proliferation, glycolysis and cell survival were mediated by Akt pathway, which was involved in cancer cell radioresistance. Shimura et al. detected that cells could acquire radioresistance causing by Akt‐mediated enhancing aerobic glycolysis.^14^ In addition, the poor radio‐response in head neck cancer cells might be also caused by up‐regulation of the Akt pathway.^22^ In our study, high expression level of *ATF3* enhanced cell radioresistance by up‐regulating pAkt while si*ATF3* could reduce it.

In spite of all the results mentioned above, there are still two major limitations to the study. First of all, the sample capacity requires further improvement. The samples were made up of 60 cases of breast cancer tissues and paracancerous tissues regardless of the patients’ age, tumour stage or if they had received adjuvant hormone or chemotherapy. To fully represent breast cancer patients, samples should be consisted of women of different races, ages, tumour stages, medical histories and so on. Secondly, other important radiation‐related signalling pathways need to be examined. For instance, researches into the function of the checkpoint kinase (CHK) signalling pathway^23^ and the Wnt/beta‐catenin signalling pathway^23^ needed to consulted so as to shed more light on how *ATF3* could be expressed in these signalling pathways to influence the effects of breast cancer radioresistance. All of the limitations would like to be involved in the next research.

In conclusion, throughout the research, *ATF3* expression could exert significant influences on various spheres of the breast cancer radioresistance that is the efficiency of radiation therapy. When *ATF3* expressed highly in breast cancer, the cancer radioresistance was enhanced and radiation therapy could not well function in causing breast cancer cell apoptosis or in remaining the G2/M phase cell cycle block. To increase the radiotherapy efficiency, methods such as silencing *ATF3* expression and inhibiting the activation of the PI3K/Akt signalling pathway were recommended.

## CONFLICT OF INTEREST

The authors declare that they have no conflict of interest.

## ETHICAL APPROVAL

All procedures performed in studies involving human participants were in accordance with the ethical standards of the institutional and/or national research committee and with the 1964 Helsinki declaration and its later amendments or comparable ethical standards.

## INFORMED CONSENT

Informed consent was obtained from all individual participants included in the study.
