# Supplementary material for: Transcription factor ATF3 mediates the radioresistance of breast cancer
Source: J Cell Mol Med. 2018 Aug 17;22(10):4664–75. doi: 10.1111/jcmm.13688 (PMC6156394; doi:10.1111/jcmm.13688)
Supplement: Supplementary file 3 [file JCMM-22-4664-s003.docx]

| **Table S1 Clinicopathological features of breast cancer patients** | | | | |
| --- | --- | --- | --- | --- |
| **Variables** | **Cases** | **ATF3** | | |
|  |  | Positive | Negative | *P* value |
| **Age** |  |  |  |  |
| ≤50 | 28 | 25 | 6 | 0.653 |
| ＞50 | 32 | 22 | 7 |  |
| **Stage** |  |  |  |  |
| Ⅰ | 15 | 9 | 7 | 0.024* |
| Ⅱ | 27 | 21 | 5 |  |
| Ⅲ | 18 | 17 | 1 |  |
| **Lymph node metastasis** |  |  |  |  |
| No | 31 | 21 | 10 | 0.039* |
| Yes | 29 | 26 | 3 |  |
| **ER status** |  |  |  |  |
| Negative | 26 | 23 | 3 | 0.096 |
| Positive | 34 | 24 | 10 |  |
| **PgR status** |  |  |  |  |
| Negative | 30 | 25 | 5 | 0.372 |
| Positive | 30 | 22 | 8 |  |
| **HER-2 status** |  |  |  |  |
| Negative | 37 | 28 | 9 | 0.526 |
| Positive | 23 | 19 | 4 |  |
| ER: estrogen receptor  PgR: progesterone receptor  HER2: human epidermal growth factor receptor 2  Data were compared by Chi-square test, *, *P*<0.05. | | | | |
