# Supplementary material for: Transcription factor ATF3 mediates the radioresistance of breast cancer
Source: J Cell Mol Med. 2018 Aug 17;22(10):4664–75. doi: 10.1111/jcmm.13688 (PMC6156394; doi:10.1111/jcmm.13688)
Supplement: Supplementary file 4 [file JCMM-22-4664-s004.docx]

**Table S2 The interference nucleotide sequences**

| Gene | Sequence (5’-3’) |
| --- | --- |
| siATF3 | CATCTTTGCCTCAACTCCAGGATTT |
| siNC | CATTTCGTCACCACTACGGATCTTT |
