# Supplementary material for: Transcription factor ATF3 mediates the radioresistance of breast cancer
Source: J Cell Mol Med. 2018 Aug 17;22(10):4664–75. doi: 10.1111/jcmm.13688 (PMC6156394; doi:10.1111/jcmm.13688)
Supplement: Supplementary file 5 [file JCMM-22-4664-s005.docx]

**Table S3 The primer sequences used in qRT-PCR**

| Gene | Sequence (5’-3’) |
| --- | --- |
| ATF3 forward | GTTTGAGGATTTTGCTAACCTGAC |
| ATF3 reverse | AGCTGCAATCTTATTTCTTTCTCGT |
| β-actin forward | GGACTTCGAGCAAGAGATGG |
| β-actin reverse | AGCACTGTGTTGGCGTACAG |
